# Supplementary material for: Tetragonal–Cubic Phase Transition and Low-Field Dielectric Properties of CH3NH3PbI3 Crystals
Source: Materials (Basel). 2021 Jul 28;14(15):4215. doi: 10.3390/ma14154215 (PMC8348982; doi:10.3390/ma14154215)
Supplement: Supplementary file 1 [file materials-14-04215-s001.zip › materials-1298429-supplementary.pdf]

Support information to the "Tetragonal-cubic phase transition and low field dielectric properties of $CH_3NH_3PbI_3$ crystals"

Roxana E. Patru[1], *Hamidreza Khassaf*[2,3], *Iuliana Pasuk*[1], *Mihaela Botea*[1], *Lucian Trupina*[1], *Constantin-Paul Ganea*[1], *Ioana Pintilie*[1], *Lucian Pintilie*[1]

[1] National Institute of Materials Physics, Atomistilor 405A, 077125 Magurele, Romania; roxana.patru@infim.ro (R.E.P.); iuliana.pasuk@infim.ro (I.P.); botea.mihaela@infim.ro (M.B.); Lucian.Trupina@infim.ro (L.T.); paul.ganea@infim.ro (P.C.G.); pintilie@infim.ro (L.P.)

[2] Department of Materials Science and Engineering , University of Connecticut, 97 North Eagleville Road, Storrs, CT 06269, USA; hamid.khassaf@gmail.com (H.K.)

[3] Institute of Materials Science, University of Connecticut, 97 North Eagleville Road, Storrs, CT 06269, USA; hamid.khassaf@gmail.com (H.K.);

* Correspondence: ioana@infim.ro; Tel.: +40-213690185

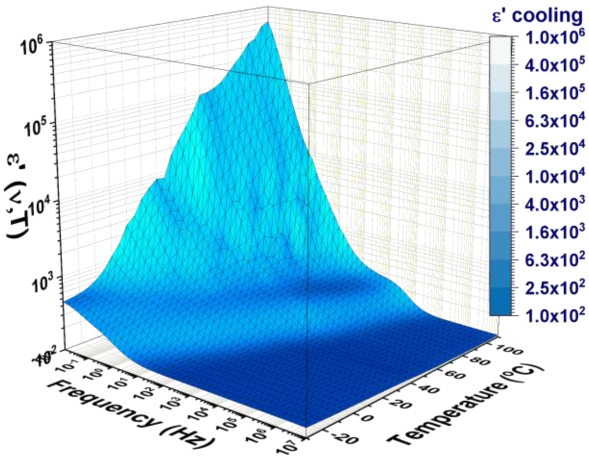

Figure S1 The real part of dielectric permittivity represented as a function of frequency and temperature, measured during cooling

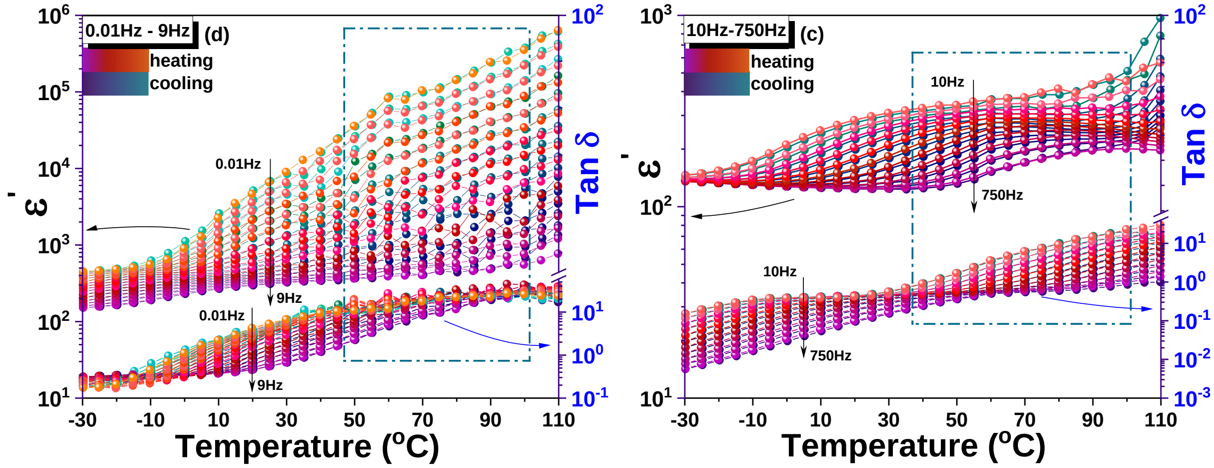

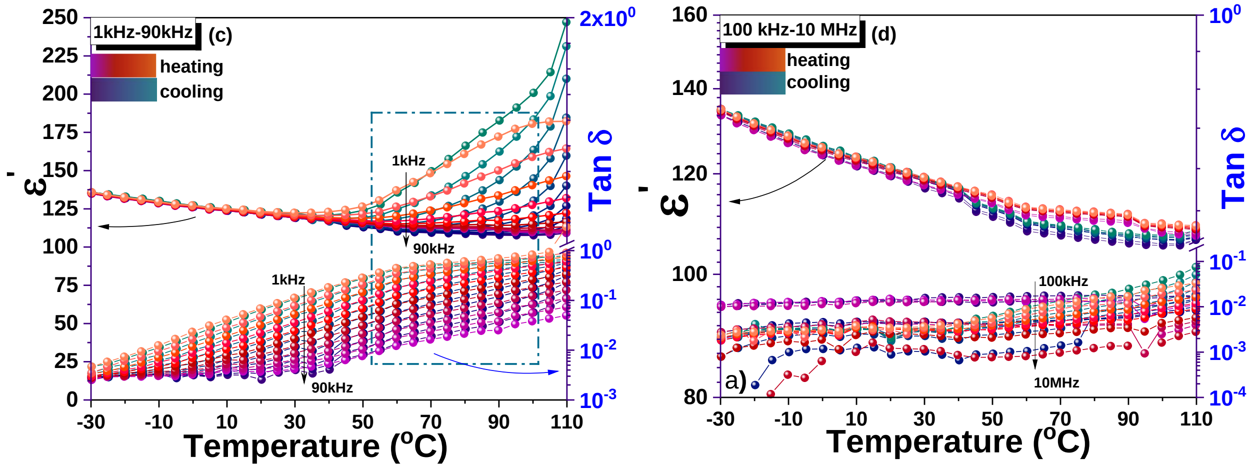

Figure S2 The temperature variation of ε′ and dielectric tangent at different frequency intervals: (a) 0.01 Hz-9 Hz; (b) 10 Hz -750 Hz; (c) 1 kHz - 90 kHz; (d) 100 kHz -10 MHz

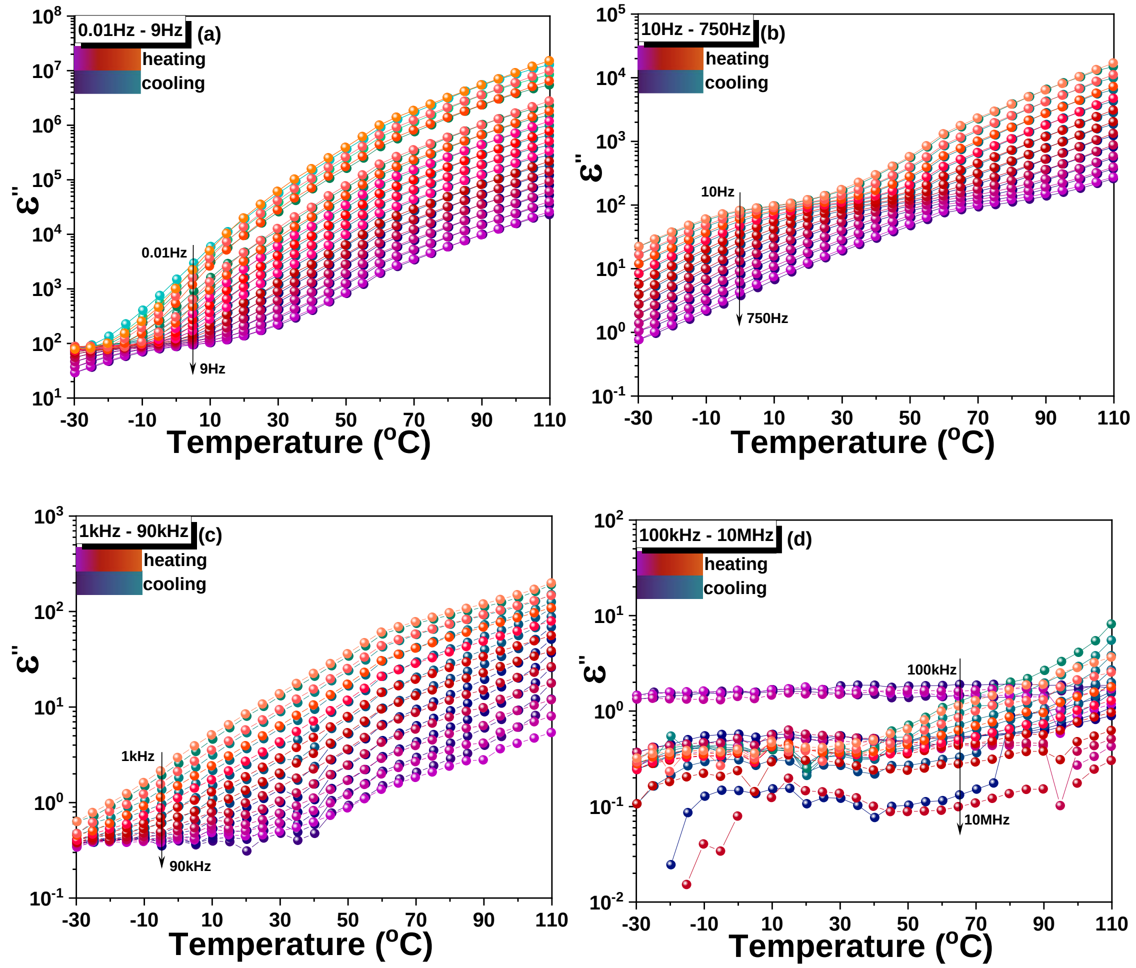

Figure S3 The temperature dependence of the loss factor ε'' measured during heating and cooling at different frequency intervals: (a) 0.01 Hz-9 Hz; (b) 10 Hz -750 Hz; (c) 1 kHz - 90 kHz; (d) 100 kHz -10 MHz

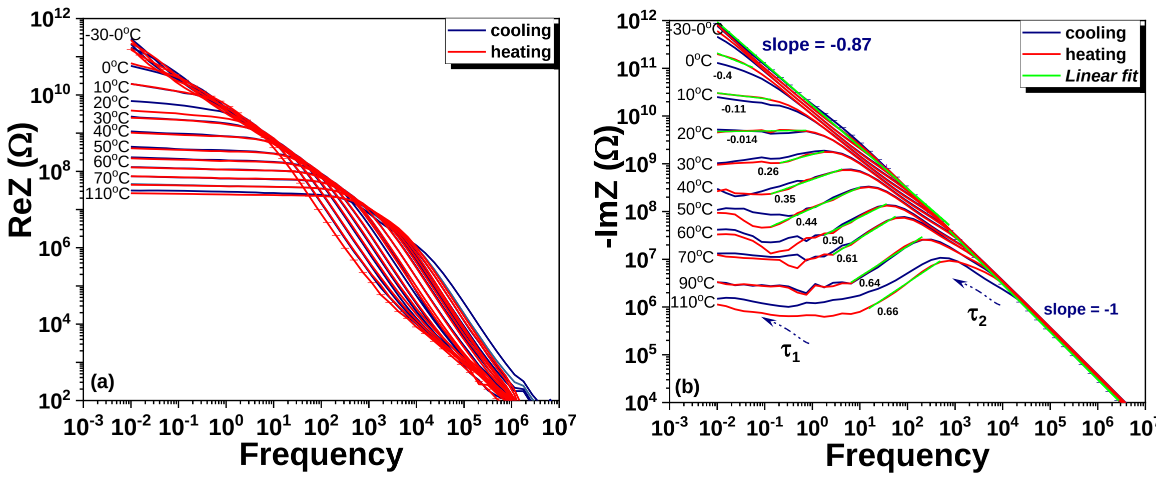

Figure S4 Frequency dependence of the complex impedance during heating and cooling down, in the temperature range of -30 °C÷110 °C :(a) the resistive real ReZ; (b) the reactive imaginary –ImZ components, (solid green lines obtained by linear fitting)

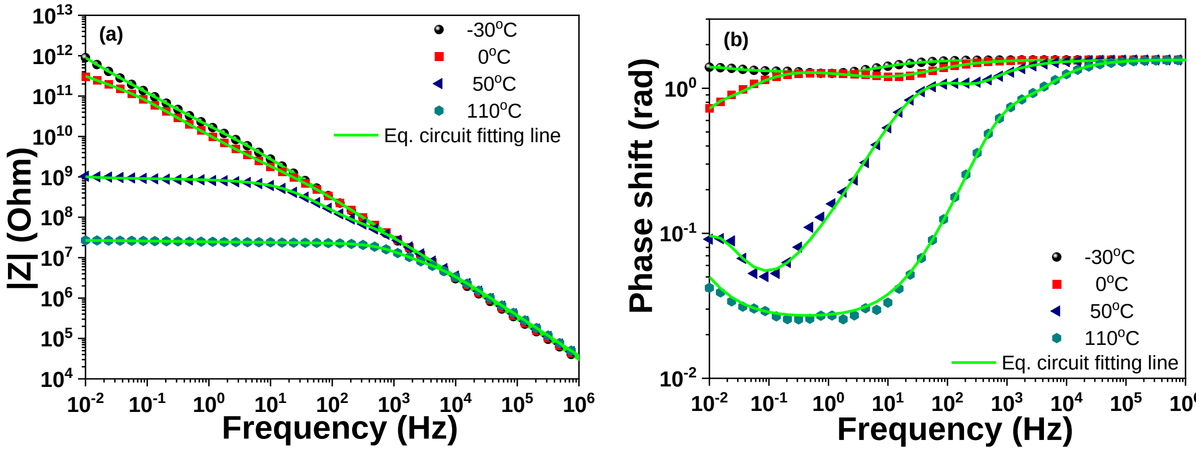

Figure S5 Impedance and phase shift fitting results obtained by using the proposed equivalent circuit: (a) Absolute impedance and (b) phase shift represented for a few specific temperatures, (solid green fitting lines obtained by using the equivalent circuit);

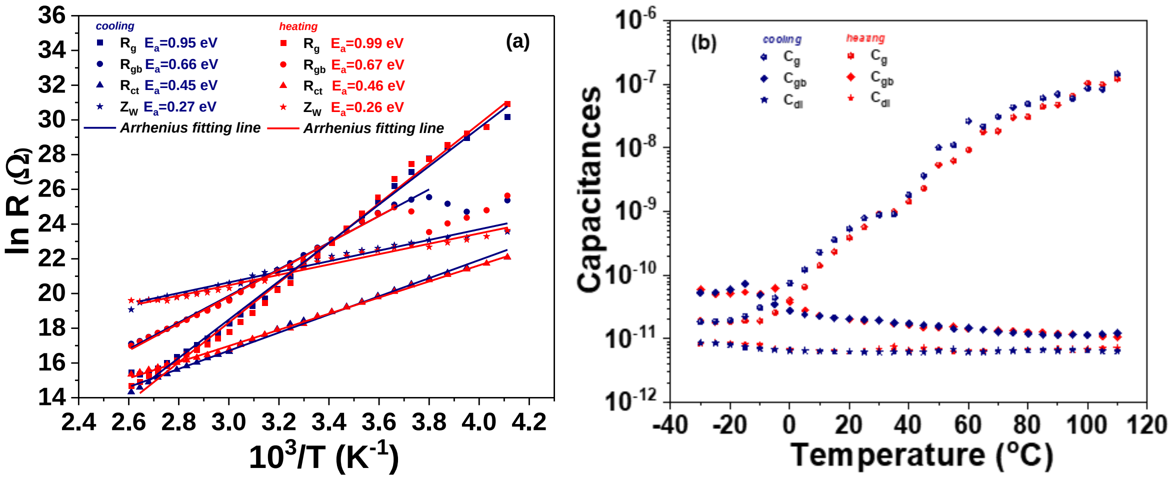

Figure S6 Temperature dependencies of the passive elements considered in the proposed equivalent circuit, during heating and cooling: (a) resistances and (b) capacitances.
